# Altered Pattern of Serum N-Glycome in Subarachnoid Hemorrhage and Cerebral Vasospasm

**DOI:** 10.3390/jcm14020465

**Published:** 2025-01-13

**Authors:** Máté Czabajszki, Attila Garami, Tihamér Molnár, Péter Csécsei, Béla Viskolcz, Csaba Oláh, Csaba Váradi

**Affiliations:** 1 Institute of Chemistry, Faculty of Materials Science and Engineering, University of Miskolc, 3515 Miskolc, Hungary; czamate@gmail.com (M.C.); bela.viskolcz@uni-miskolc.hu (B.V.); 2 Department of Neurosurgery, Borsod-Abaúj-Zemplén County Center Hospital and University Teaching Hospital, 3526 Miskolc, Hungary; olahcs@gmail.com; 3 Institute of Energy, Ceramic and Polymer Technology, University of Miskolc, 3515 Miskolc, Hungary; attila.garami@uni-miskolc.hu; 4 Department of Anesthesiology and Intensive Care, University of Pécs Medical School, 7624 Pécs, Hungary; tihamermolnar@yahoo.com (T.M.); csecsei.peter@pte.hu (P.C.); 5 Mathias Institute, University of Tokaj, 3950 Sárospatak, Hungary

**Keywords:** serum glycosylation, liquid chromatography, mass spectrometry, subarachnoid hemorrhage, cerebral vasospasm

## Abstract

**Background**: Subarachnoid hemorrhage is a serious condition caused by ruptured intracranial aneurysms, resulting in severe disability mainly in young adults. Cerebral vasospasm is one of the most common complication of subarachnoid hemorrhage; thus, active prevention is key to improve the prognosis. The glycosylation of proteins is a critical quality attribute which is reportedly altered in patients diagnosed with acute ischemic stroke. In this study, we examined the N-glycosylation profile of serum glycoproteins in patients with subarachnoid hemorrhage without vasospasm compared to patients with vasospasm. **Methods**: The serum N-glycans were released by PNGase F (Peptide: N-glycosidase F) digestion and subsequently labeled by procainamide via reductive amination. The samples were analyzed by hydrophilic-interaction liquid chromatography after solid-phase extraction-based sample purification. **Results**: Besides the glycosylation pattern, we also investigated the biomarkers following subarachnoid hemorrhage. Multiple statistical analyses were performed in order to find significant differences and identify potential prediction factors of cerebral vasospasm. Significant differences were identified such as higher sialylation on bi-, tri-, and tetra-antennary structures in patients with subarachnoid hemorrhage and cerebral vasospasm. **Conclusions**: Our results suggest that glycosylation analysis can improve the identification of patients with cerebral vasospasm in combination with laboratory parameters.

## 1. Introduction

Subarachnoid hemorrhage (SAH) caused by ruptured cerebral aneurysm is a hemorrhagic type of stroke disease [1]. Unruptured aneurysms affect about 3.2% of people worldwide while ruptured aneurysms occur in approximately 10 per 100,000 cases [2]. The main risk factors in the development of SAH are elevated blood pressure, smoking, taking oral contraceptives, and pregnancy [3]. The occurrence of rupture for unruptured aneurysms is approximately 0.5–0.6/year, mainly depending on the location, risk factors, and size of the aneurysms [4]. The annual incidence of subarachnoid hemorrhage is 10–13/100,000 people, with a high rate of morbidity and mortality: 15–20% of patients usually die at the scene or before reaching the hospital [5]. The mortality rate of aneurysmal subarachnoid hemorrhage within 72 h is ~20%, and according to international data, the overall first month mortality can reach nearly 50% [6]. In aneurysmal subarachnoid hemorrhage, cerebral vasospasm (CVS) is one of the most common complications, often leading to a mortality of 6–8 percent [7]. Consequential cerebral ischemia can cause severe chronic neurological disabilities in about 10 percent of cases [8]. Vasospasms usually manifest on the 4th day post-onset, peaking around the 7th or 8th day, and then the value of the spasm continuously decreases [9]. The risk of vasospasm occurrence depends on the initial neurological state (Hunt–Hess score) and the magnitude/extension of the subarachnoid blood, which are characterized by the Fischer scale [10]. The diagnosis of the vasospasm is confirmed by scan examinations to identify the diameter of the arteries, mainly using transcranial Doppler ultrasound (TCD), which is an easy, non-invasive bedside examination [11]. As a physical rule, the higher the velocity of the blood stream, the more superior the value of the vasospasm, which can be detected via TCD [12]. The application of digital subtraction angiography (DSA), computed tomography angiography (CTA), and magnetic resonance angiography (MRA) is also able to provide further and more precise information about the diameter and the alteration of the cerebral blood supply [13]. Concerning the secondary vasospasm that develops in patients who survive the initial brain hemorrhage, there are currently no known laboratory, clinical, instrumental, or other parameters that correlate with the likelihood of vasospasm development in the pre-spasm period (0–4 days) before the clinical and radiological detection of the vasospasm. The requirement of specific diagnostic procedures in the identification of vasospasms highlights the need of novel biomarkers to improve early detection [14]. Glycosylation is a post-translational modification of proteins reportedly altered in multiple diseases including peripheral artery diseases [15], acute ischemic stroke [16], and other neurological disorders [17]. N-glycosylation is the attachment of a carbohydrate molecule to a parent protein at the nitrogen atom of the amino group of asparagine via a β1-glycosidic bond. N-glycan structures consist of a core glycan structure, containing two GlcNAc monosaccharides and three mannose (Man) residues. Additional monosaccharides can be connected to this core structure, creating highly branched and complex structures. One of the main difficulties in the quantitation of glycan structures is the need of sample derivatization due to the lack of a fluorophore group. This is most commonly performed by reductive amination, resulting in the attachment of a fluorescent dye to each glycan structure. The possible presence of multiple antennae on glycan species necessitates the use of high-resolution separation techniques, most commonly hydrophilic-interaction liquid chromatography (HILIC-UPLC). The clinical prediction of the development of CVS based on laboratory parameters and medical examination is currently uncertain after SAH. The goal of this research was to identify the alterations of serum N-glycome in patients diagnosed with SAH and CVS compared to healthy controls (HC).

## 2. Materials and Methods

### 2.1. Chemicals

All chemicals used in these experiments, including acetonitrile, formic acid, and procainamide, were obtained from Sigma-Aldrich (St. Louis, MO, USA). The enzyme for the glycan release was purchased from New England Biolabs (Ipswich, MA, USA).

### 2.2. Patient Samples

Serum samples from 22 healthy controls, 22 with SAH, and 22 with vasospasm confirmed by TCD were collected at the Borsod Academic County Hospital (Miskolc, Hungary). All patients signed informed consent forms in accordance with the Declaration of Helsinki. This research was approved by the Regional Research Ethics Committee (IG-102102/2018).

### 2.3. N-Glycan Sample Preparation

PNGase F enzymatic digestion was applied on each patient sample in order to release N-linked carbohydrates using the New England Biolabs (Ipswich, MA, USA) deglycosylation protocol. Briefly, 9 μL of serum sample was denatured by the addition of 1 μL 10× denaturation buffer and incubated for 15 min at 65 °C. This was followed by the addition of 7 μL ultrapure water, 2 μL of 10× glycobuffer, and 1 μL of PNGase F. The mixture was incubated overnight at 37 °C. The fluorescent derivatization of the liberated oligosaccharides was performed using 10 μL of labeling solution (0.3 M procainamide and 300 mM picoline borane in 70%/30% of dimethyl sulfoxide/acetic acid) for 4 h at 65 °C. Sample clean-up was obtained by solid-phase extraction columns (GL Sciences Inc., Tokyo, Japan) according to the manufacturer’s protocol.

### 2.4. UPLC-FLR-MS Analysis

N-glycomics analysis was performed by a Waters Acquity H-class liquid chromatography instrument with fluorescence mass spectrometric detection controlled by MassLynx 4.2 (Waters, Milford, MA, USA). In each chromatographic separation a Waters BEH Glycan column, 100 × 2.1 mm i.d., 1.7 μm, was used at 60 °C, with 50 mM ammonium formate (pH 4.4) and acetonitrile. In total, 5 μL of sample was injected in all runs while the temperature of the sample manager was set at 15 °C. The fluorescence excitation and emission wavelengths were λ_ex_ = 308 nm and λ_em_ = 359 nm. In the MS setup, the electrospray voltage was 2.2 kV in positive mode while the desolvation temperature was 120 °C with 800 L/h desolvation gas flow. MS data were obtained over the range of 600–2500 *m*/*z* and the MS/MS fragmentation was performed by a ramp of 30 to 60 kV collision energy.

### 2.5. Data Analysis

Chromatographic data integration was carried out in Empower 3 chromatography software (Waters, Milford, MA, USA). The calculation of the glycan *m*/*z* values was performed in GlycoWorkBench 2.0. Glycan nomenclature was used as is visualized in Supplementary Figure S1 and has been described by Harvey et al. [18]. Past 4.11 software was used to evaluate the linear discriminant analysis (LDA) and IBM SPSS Statistics 25 to perform Kruskal–Wallis tests and Mann–Whitney pairwise comparisons.

## 3. Results

**Figure 1:**
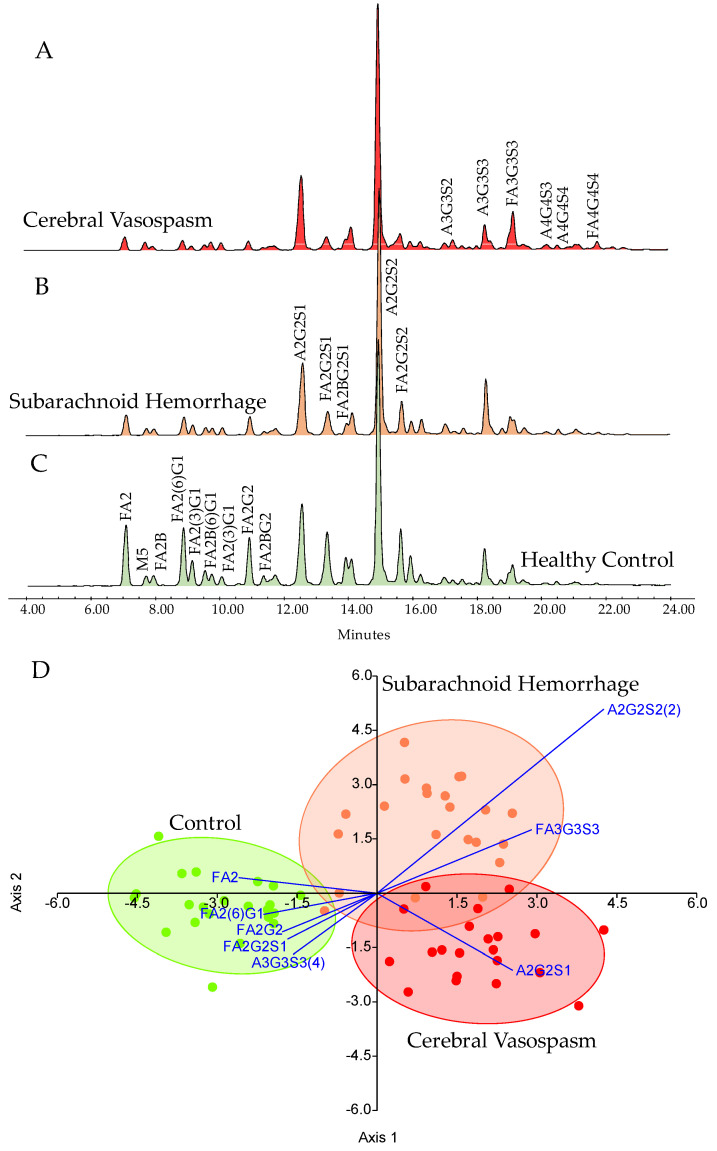
Representative HILIC-UPLC-FLR chromatograms of the analyzed patient groups (**A**–**C**), showing a lower level of neutral structures (FA2, FA2G1, and FA2G2) and their linear discriminant analysis based on the glycosylation pattern (**D**).

**Figure 2:**
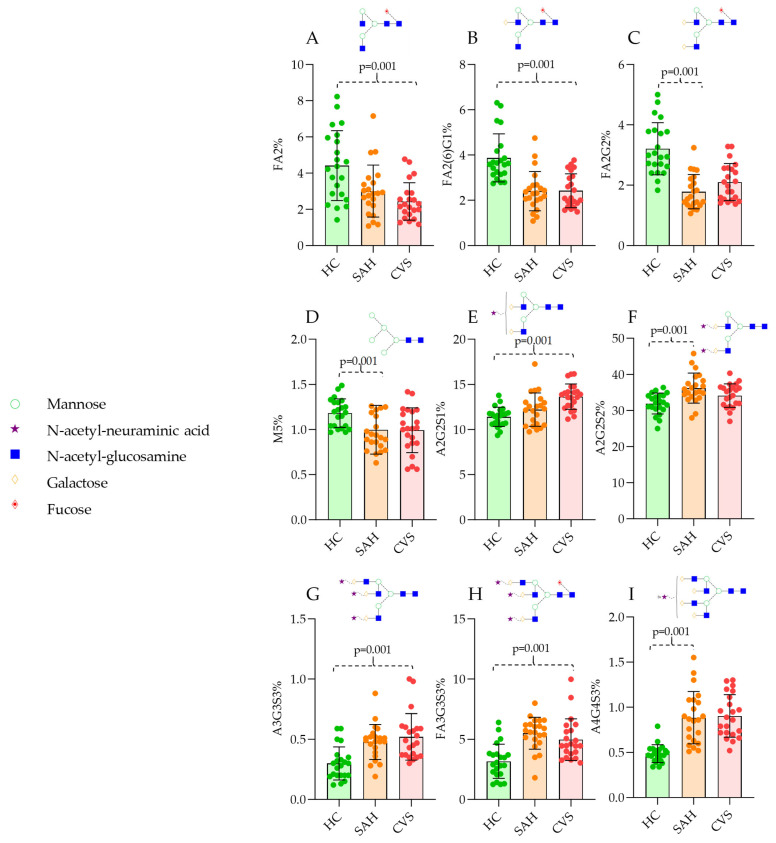
Significantly different N-glycan ratios of SAH and CVS patients compared to healthy controls. (**A**): FA2, (**B**): FA2G1, (**C**): FA2G2, (**D**): M5, (**E**): A2G2S1, (**F**): A2G2S2, (**G**):A3G3S3, (**H**): FA3G3S3, (**I**): A4G4S3.

In total, 22 HC, 22 SAH, and 22 CVS serum samples were analyzed by HILIC-FLR-MS. Representative chromatograms are visualized in Figure 1 with the main structures of the corresponding peaks. The quantitation of the fluorescently derivatized glycan structures was evaluated based on the fluorescence spectra, analyzing 36 peaks. Each glycan structure was identified by mass spectrometric analysis using their mass-to-charge ratio. The average area% of each identified glycan is listed in Supplementary Table S1, where 24 structures were found to be significantly different using the Kruskal–Wallis test. As is visualized in Figure 1, clear differences were identified in both SAH and CVS patients compared to HC. Using linear discriminant analysis, each patient group was separated based on their glycosylation pattern, with a slight overlap between SAH and CVS (Figure 1D). The main neutral structures including FA2 (fucosylated bi-antennary structure), FA2G1 (fucosylated and mono-galactosylated bi-antennary structure), and FA2G2 (fucosylated and bi-galactosylated bi-antennary structure) contributed to the discrimination of HC group, while sialylated structures such as A2G2S1 (mono-sialylated bi-antennary structure), A2G2S2 (bi-sialylated bi-antennary structure), and FA3G3S3 (fucosylated and tri-sialylated tri-antennary structure) provided the separation of SAH and CVS group. Most of these structures were also found to be significantly different between HC and the patient groups, with a main trend of lower levels of neutral structures and higher sialylation in diseased patients (Figure 2). These findings are supported by previous reports where protein glycosylation was found to be critical in other circulatory disorders, affecting the outcome of the stroke [16].

**Figure 3:**
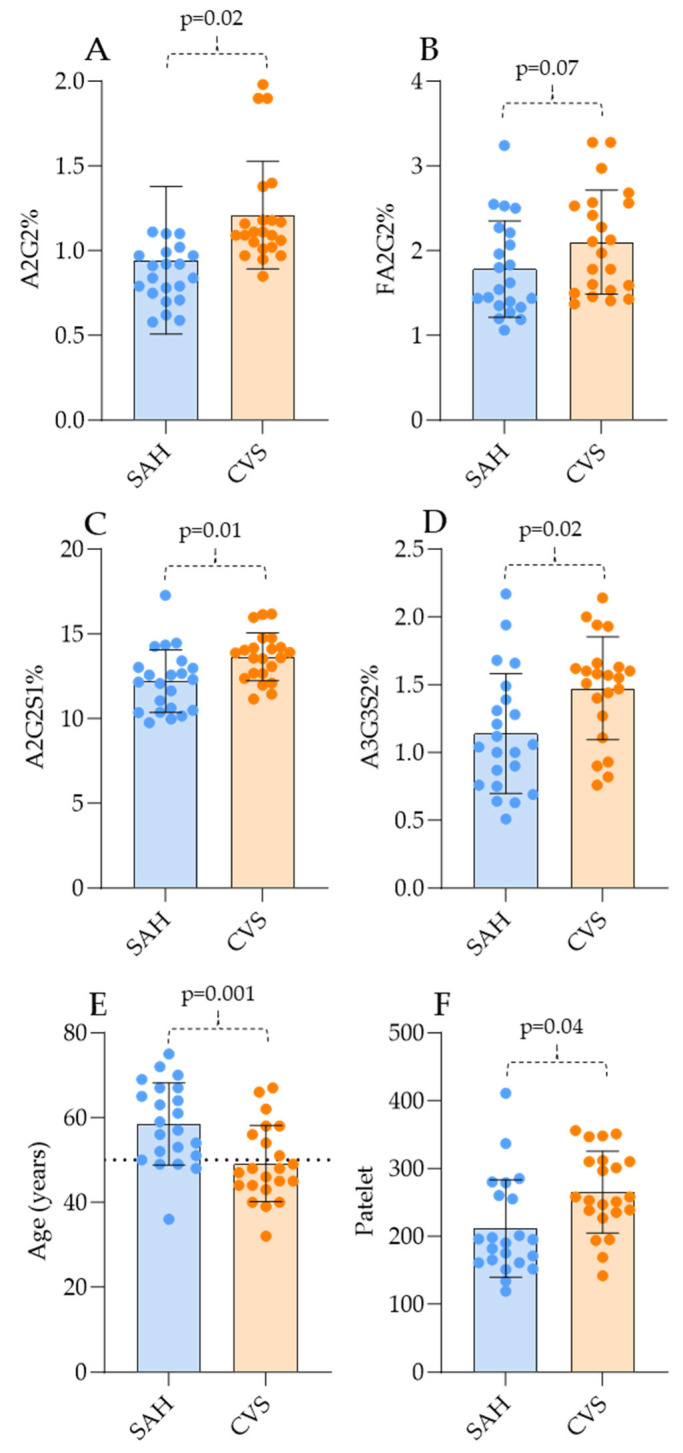
The most significant N-glycan levels and laboratory data of SAH and CVS patients. (**A**): A2G2, (**B)**: FA2G2, (**C**): A2G2S1, (**D**): A3G3S2, (**E**)**:** Age, (**F**): Platelet count.

While the evidence of significant differences in the glycosylation pattern of SAH and CVS patients was clear, one of our main goals was to examine the possibility of discriminating between SAH and CVS patients. Comparing the serum N-glycome pattern, four main significant differences were identified by the Mann–Whitney test, namely, higher levels of A2G2, FA2G2, A2G2S1, and A3G3S2 (Figure 3).

To establish an order of importance in the obtained glycosylation data and laboratory parameters, point-biserial correlation was performed between SAH and CVS patients. Similarly to the Mann–Whitney pairwise comparison, age was one of the most critical parameters which could be associated with the development of CVS, while the level of A2G2S1 was also significant (Supplementary Table S1). ROC curve analysis was performed using the most significant differences in order to identify the diagnostic value of the revealed differences. The levels of A2G2, FA2G2, A2G2S1, and A2BG3S2 and platelet count showed a high predictive value (>0.7) in the development of CVS, alongside younger age, as is shown in Supplementary Figure S2.

## 4. Discussion

Altered protein glycosylation has been described before in several circulatory disorders such as acute ischemic stroke [19] and peripheral artery diseases [15]. The glycosylation of immunoglobulin G was found to be associated with the main risk factors of ischemic stroke such as age, obesity, dyslipidemia, and hypertension [15]. Glycosylated hemoglobin A1 was reported as a predictive factor in the development of symptomatic hemorrhage after thrombolysis for acute stroke [20]. Changes in plasma sialic acid levels and sialidase activity were also identified as potential markers in acute stroke [21]. Analyzing the laboratory data, age and platelet number were significantly different between the SAH and CVS groups. Based on our data, the development of CVS is more frequent in patients under 50 years of age, which is in perfect agreement with previous reports where the association of age and vasospasm has been described [22]. It was also published that age <50 includes a 5-fold risk of CVS occurrence, which also supports our results [22]. Platelet number was found to be significantly higher in CVS patients, as is shown in Figure 2F, although this is in contrast with some studies where a lower platelet count was found in CVS patients [23,24]. This can be due to the fact that high platelet aggregability has also been described in patients with symptomatic vasospasm over time, although our samples were taken on the first day of CVS formation, suggesting that CVS activates platelets and promotes thrombus formation, thus decreasing the free platelet count over time [25]. These are important findings as platelets play crucial roles in hemostasis, inflammation, and the regulation of vascular tone [26]. They contribute to clot formation through self-aggregation, interaction with fibrin, and fibrinogen and endothelial adhesion [27]. Additionally, they are involved in inflammatory responses by releasing chemokines and cytokines and interacting with immune cells [28]. Platelets also influence blood vessel constriction by secreting vasoconstrictive agents [29]. The main limitations of this study are the small sample size and the slight overlap between the SAH and CVS patient groups, which restrict the robustness of the findings. The scientific knowledge on glycosylation alterations in SAH and CVS is also incomplete as this is one of the first glycomics studies in these conditions.

## 5. Conclusions

In this study, we analyzed the serum N-glycome of healthy, vasospasmotic, and non-vasospasmotic patient groups. Significant differences were identified in both glycosylation data and laboratory parameters. Higher sialylation, younger age, and increased platelet count were found to be the most critical factors in the development of vasospasms. Our future plan is to examine the correlation of laboratory parameters and serum N-glycome with the appearance of vasospasms via machine learning techniques.

## Data Availability

The data presented in this study are available on request from the corresponding author.

## Supplementary Materials

The following supporting information can be downloaded at: https://www.mdpi.com/article/10.3390/jcm14020465/s1. Table S1. Relative peak area percentages in HC, SAH and CVS patients, Table S2. Point biseral correlation of laboratory parameters and N-glycomic data, Figure S1. Schematic representation of the nomenclature of glycan structures, Figure S2. ROC curve analysis of SAH and CVS patients using the most significant differences
