# Supplementary material for: Altered Pattern of Serum N-Glycome in Subarachnoid Hemorrhage and Cerebral Vasospasm
Source: J Clin Med. 2025 Jan 13;14(2):465. doi: 10.3390/jcm14020465 (PMC11765641; doi:10.3390/jcm14020465)

Supplementary

Altered Pattern of Serum N-Glycome in Subarachnoid Hemorrhage and Cerebral Vasospasm

Máté Czabajszki [1,2], Attila Garami [3], Tihamér Molnár [4], Péter Csécsei [4], Béla Viskolcz [1], Csaba Oláh [2,5] and Csaba Váradi [1,*]

[1] Institute of Chemistry, Faculty of Materials Science and Engineering, University of Miskolc, 3515 Miskolc, Hungary; czamate@gmail.com (M.C.); bela.viskolcz@uni-miskolc.hu (B.V.)
[2] Department of Neurosurgery, Borsod-Abaúj-Zemplén County Center Hospital and University Teaching Hospital, 3526 Miskolc, Hungary; olahcs@gmail.com
[3] Institute of Energy, Ceramic and Polymer Technology, University of Miskolc, 3515 Miskolc, Hungary; attila.garami@uni-miskolc.hu
[4] Department of Anesthesiology and Intensive Care, University of Pécs Medical School, 7624 Pécs, Hungary; tihamermolnar@yahoo.com (T.M.); csecsei.peter@pte.hu (P.C.)
[5] Mathias Institute, University of Tokaj, 3950 Sárospatak, Hungary
* Correspondence: csaba.varadi@uni-miskolc.hu; Tel.: +36-30-894-7730

Supplementary Table S1. Relative peak area percentages in HC, SAH and CVS patients

	Control	SD	SAH	SD	CVS	SD	p value
FA2	4.42	1.93	3.01	1.43	2.44	1.25	0.00
M5	1.18	0.16	1.00	0.27	0.99	0.27	0.00
FA2B	1.12	0.47	0.81	0.32	0.68	0.30	0.00
FA2(6)G1	3.87	1.06	2.40	0.87	2.42	0.82	0.00
FA2(3)G1	1.72	0.46	1.04	0.49	1.03	0.40	0.00
FA2BG1	1.32	0.24	1.00	0.30	0.98	0.29	0.00
M6	1.28	0.19	1.15	0.34	1.12	0.29	0.01
A2G2	0.94	0.18	0.94	0.44	1.21	0.40	0.00
FA2G2	3.21	0.87	1.78	0.57	2.10	0.63	0.00
FA2BG2	0.66	0.18	0.49	0.21	0.54	0.19	0.00
FA2FG2	0.34	0.16	0.40	0.17	0.36	0.15	0.37
M4G1S1	1.30	0.26	1.08	0.29	1.06	0.30	0.01
A2G2S1	11.40	1.07	12.21	1.85	13.65	2.39	0.00
FA2G2S1	4.97	1.15	3.73	0.74	4.05	0.94	0.00
FA2BG2S1	2.23	0.52	1.93	0.95	1.92	0.86	0.02
A2G2S2(1)	2.89	1.07	3.01	0.91	2.89	0.82	0.89
A2G2S2(2)	31.96	2.89	36.22	4.17	34.15	5.92	0.00
FA2G2S2(1)	0.49	0.13	0.52	0.14	0.53	0.13	0.43
FA2G2S2(2)	4.21	0.93	3.95	0.61	3.90	0.80	0.44
FA2BG2S2	2.10	1.01	1.77	0.48	1.86	0.57	0.63
A2BG3S2(1)	1.44	0.53	1.14	0.44	1.47	0.45	0.04
A2BG3S2(2)	0.26	0.05	0.39	0.10	0.36	0.10	0.00
A2BG3S2(3)	1.49	0.36	1.32	0.35	1.64	0.38	0.01
FA3G3S2	0.82	0.33	1.33	0.36	1.44	0.42	0.00
A3G3S3(1)	0.69	0.31	0.56	0.21	0.60	0.18	0.27

A3G3S3(2)	0.18	0.07	0.28	0.14	0.27	0.13	0.00
A3G3S3(3)	0.30	0.14	0.48	0.15	0.52	0.17	0.00
A3G3S3(4)	5.00	1.99	3.56	1.49	4.05	1.34	0.02
A4G4S2	0.56	0.13	0.95	0.30	0.83	0.29	0.00
A3G3S3(5)	1.62	0.59	1.73	0.56	1.66	0.53	0.81
FA3G3S3	3.17	1.42	5.50	1.33	4.98	1.62	0.00
A4G4S3(1)	0.81	0.21	0.83	0.26	0.93	0.27	0.22
A4G4S3(2)	0.49	0.10	0.89	0.29	0.90	0.27	0.00
A4G4S4(1)	0.44	0.27	0.51	0.25	0.56	0.21	0.10
A4G4S4(2)	0.53	0.20	0.53	0.20	0.62	0.23	0.31
A4G4S4(3)	0.16	0.07	0.47	0.24	0.37	0.22	0.00
FA4G4S4	0.45	0.19	1.12	0.48	0.91	0.42	0.00

Supplementary Table S2. Point biseral correlation of laboratory parameters and N-glycomic data

Peak	Point Biseral	p-value	Accepted	abs
Age	-0.50	0.00	1	0.5
A2BG3S2(3)	0.46	0.00	1	0.46
A2G2S1	0.42	0.01	1	0.42
A2BG3S2(1)	0.36	0.02	1	0.36
A2G2	0.35	0.02	1	0.35
Platelet	0.32	0.04	1	0.32
FA2G2	0.29	0.07	1	0.29
Neutrofil	0.26	0.11	1	0.26
A2G2S2(2)	-0.26	0.10	1	0.26
FA4G4S4	-0.25	0.12	1	0.25
FA2	-0.24	0.14	1	0.24
WBC	0.23	0.14	1	0.23
FA2B	-0.23	0.15	1	0.23
K	0.21	0.18	1	0.21
A4G4S4(3)	-0.21	0.19	1	0.21
FA2G2S1	0.2	0.20	1	0.2
A4G4S2	-0.2	0.20	1	0.2
A4G4S3(1)	0.19	0.24	1	0.19
A2BG3S2(2)	-0.18	0.27	1	0.18
FA3G3S3	-0.17	0.29	1	0.17
A3G3S3(4)	0.16	0.33	1	0.16
A4G4S4(2)	0.16	0.32	1	0.16
FA3G3S2	0.15	0.36	1	0.15
Karbamid	-0.15	0.36	1	0.15
CRP	-0.15	0.36	1	0.15
FA2BG2	0.14	0.38	1	0.14
A3G3S3(3)	0.14	0.37	1	0.14
Lymphocyte	-0.13	0.41	1	0.13

Supplementary Figure S1. Schematic representation of the nomenclature of glycan structures

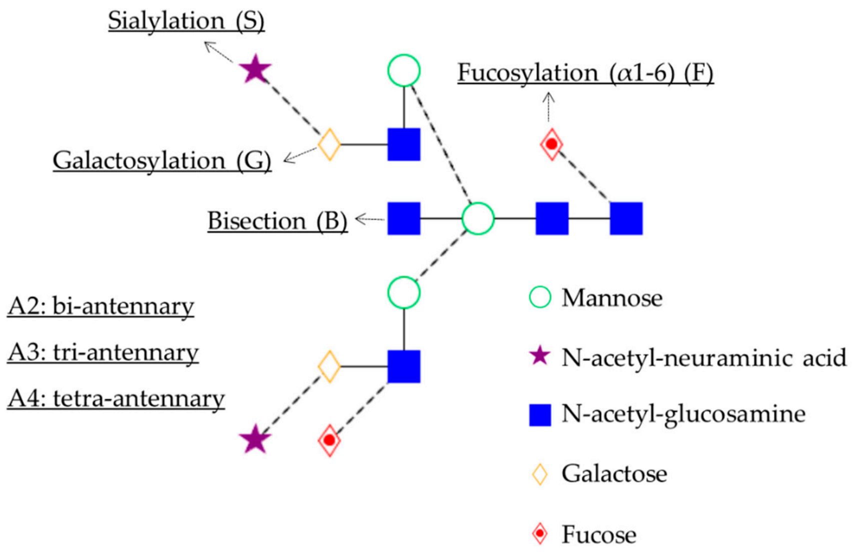

Supplementary Figure S2. ROC curve analysis of SAH and CVS patients using the most significant differences

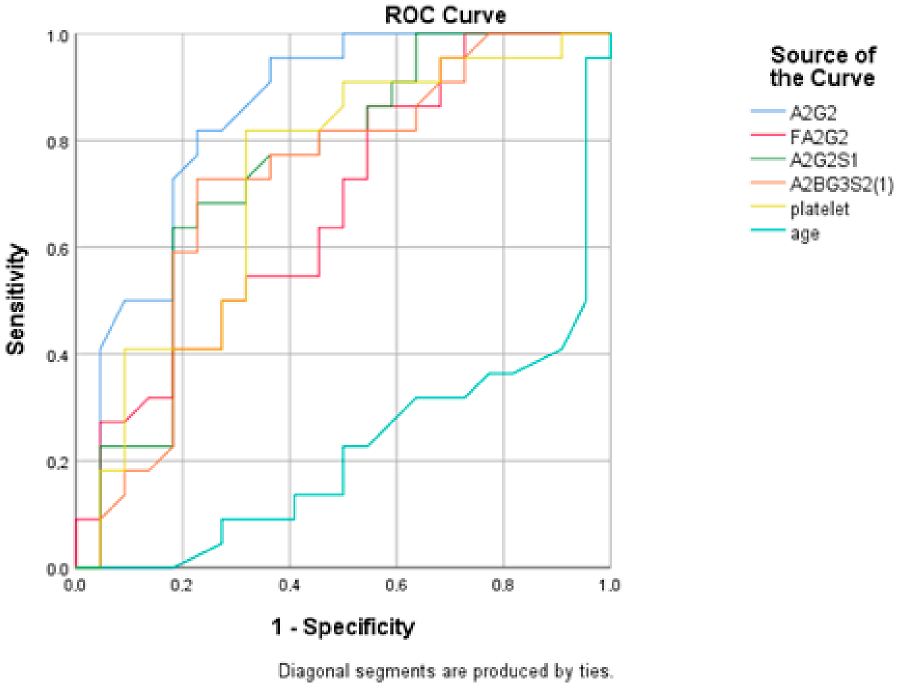

Supplement: Supplementary file 1 [file jcm-14-00465-s001.zip › jcm-3354503-supplementary.pdf]
